# The impact of nanoparticle-driven lysosomal alkalinization on cellular functionality

**DOI:** 10.1186/s12951-018-0413-7

**Published:** 2018-10-31

**Authors:** Bella B. Manshian, Suman Pokhrel, Lutz Mädler, Stefaan J. Soenen

**Affiliations:** 1 NanoHealth and Optical Imaging Group, Department of Imaging and Pathology, KU Leuven, Leuven, Belgium; 2 Molecular Small Animal Imaging Center, KU Leuven, Leuven, Belgium; 3 Foundation Institute of Materials Science (IWT), Department of Production Engineering, University of Bremen, 28359 Bremen, Germany; 4 Leibniz Institute for Materials Engineering IWT, Badgasteiner Str. 3, 28359 Bremen, Germany

**Keywords:** Nanotoxicity, Nanomedicine, Gold nanoparticles, Silicon dioxide nanoparticles

## Abstract

**Background:**

The biomedical use of nanosized materials is rapidly gaining interest, which drives the quest to elucidate the behavior of nanoparticles (NPs) in a biological environment. Apart from causing direct cell death, NPs can affect cellular wellbeing through a wide range of more subtle processes that are often overlooked. Here, we aimed to study the effect of two biomedically interesting NP types on cellular wellbeing.

**Results:**

In the present work, gold and SiO_2_ NPs of similar size and surface charge are used and their interactions with cultured cells is studied. Initial screening shows that at subcytotoxic conditions gold NPs induces cytoskeletal aberrations while SiO_2_ NPs do not. However, these transformations are only transient. In-depth investigation reveals that Au NPs reduce lysosomal activity by alkalinization of the lysosomal lumen. This leads to an accumulation of autophagosomes, resulting in a reduced cellular degradative capacity and less efficient clearance of damaged mitochondria. The autophagosome accumulation induces Rac and Cdc42 activity, and at a later stage activates RhoA. These transient cellular changes also affect cell functionality, where Au NP-labelled cells display significantly impeded cell migration and invasion.

**Conclusions:**

These data highlight the importance of in-depth understanding of bio-nano interactions to elucidate how one biological parameter (impact on cellular degradation) can induce a cascade of different effects that may have significant implications on the further use of labeled cells.

## Background

The biological behavior of nanoparticles (NPs) is currently receiving much attention, in particular to enhance our understanding of any potential hazards involved in NP exposure and to optimize the use of nanotechnology in biomedical applications [1–3]. Most studies to date involve the use of cell cultures as a good model system that can provide in-depth mechanistic insight into the precise nature of how the cells interact with the engineered NPs [4]. Other advantages of using cell culture models are the need for less animal studies which greatly enhances the speed with which the assays can be performed, while also reducing the number of animals required for in vivo studies. Novel technologies are being implemented to further increase the capacity to perform nanotoxicological research at high speeds, including automated high-content imaging, transcriptomics and proteomics [5–8].

The big efforts made have generated large amounts of data, which can be used to decipher the precise mechanisms by which NPs interact with their biological environment [9–13]. The wide variety in different types of NPs and conditions used for exposure of the NPs to their biological environment results in the generation of highly specific data that is relevant to a particular NP formulation used under very specific conditions. Although these specific mechanisms are very interesting and need to be investigated, more emphasis has recently been put on large-scale comparative studies of highly similar NP formulations [9]. These studies either enable researchers to link particular biological effects to one single NP-associated parameter [14], or define new general paradigms by which NPs can affect biological systems [15].

Based on the data obtained, several paradigms have been defined which appear to be vital in how the cell reacts to the presence of any NPs. The generation of oxidative stress has been shown to be involved in most types of NPs among a wide array of cell types [16]. As different cell types have different levels of natural antioxidants such as glutathione to defend themselves against the damages incurred from elevated levels of reactive oxygen species (ROS) [17], any elevation in ROS does not immediately result in cell death, depending on the extent of ROS produced and the nature of the cell type used [17]. A second paradigm lies in the possible biodegradation of the NPs when subjected to the degradative microenvironment of the cellular endosomal network [18]. Several types of NPs (e.g. ZnO, CuO, Ag) have shown to display pH-dependent dissolution properties and when internalized by the cells through endocytosis, the acidic endosomal lumen can promote NP degradation [19, 20]. The degradation is then linked to the release of potentially toxic metal ions, which can cause cell death [6, 19, 20]. It remains somewhat a matter of debate to what extent any observed effects are either due to the NPs themselves, the metal ions already present in the extracellular medium due to pre-dissolution of the NPs at neutral pH, or the metal ions released intracellularly after cellular NP uptake [6]. In most cases, all three components will contribute to the observed cellular effects, but intracellularly released ions have been suggested to locally reach high levels which can exceed toxic thresholds and hereby induce cellular damage at levels where free metal ions that distribute more homogenously do not cause such effects [6].

A third paradigm is the disturbance of cellular autophagy levels through NP exposure [15]. The precise nature of this effect remains rather unclear and is the topic of interest in a wide number of studies [21–23]. Initially, several groups suggested that a large number of NPs were capable of inducing autophagy and result in so-called autophagic cell death [24, 25]. Although cell death through autophagy-related mechanisms is possible, the autophagy community has labelled autophagic cell death a misnomer [26], as it was often based on the wrong interpretation of results. Autophagy is primarily a self-protective mechanism, where any cell that is undergoing stress resulting in damaged organelles (e.g. mitochondria) can result in the induction of autophagy to clear the damaged organelles and recycle their constituents for other cellular processes [27]. As such, any dead cell can show signs of elevated autophagy as a secondary effect as the cell was simply trying to defend itself and recover from the damage to which it finally succumbed. More recent studies have however shown that high levels of autophagy can result in cell death as the cells is literally “eating itself” [27]. From the NP community, similar confusing findings have been reported, where in most studies it has been shown that NPs cause cell death through autophagy, while several other studies have reported the induction of autophagy as a cellular defense mechanism to cope with the NPs [28, 29]. Any such effects may be in part explained by differences in cellular NP levels, where initially, autophagy induction may be self-protective and inhibit apoptotic signaling, while at higher levels, apoptosis itself can result in cell death [5]. Another point of confusion lies in whether autophagy is actively induced [30] or whether it is related to an accumulation of autophagosomes due to a reduced clearance by lysosomes [31]. Although autophagy disturbances have been associated to a wide variety of NPs [15], it is not yet considered a general mechanism either.

Autophagy is a cellular degradation mechanism and may come into play when the overall degradative capacity of the cell is reduced [27]. In the present work, we employ gold and silicon dioxide NPs of similar size and surface charge as models agents to study the contribution of the composition of NP cores on their cellular effects, with a special emphasis on any disturbances in the capacity of cellular degradation and associated signaling.

## Results and discussion

### Nanoparticle characterization

**Fig. 1:**
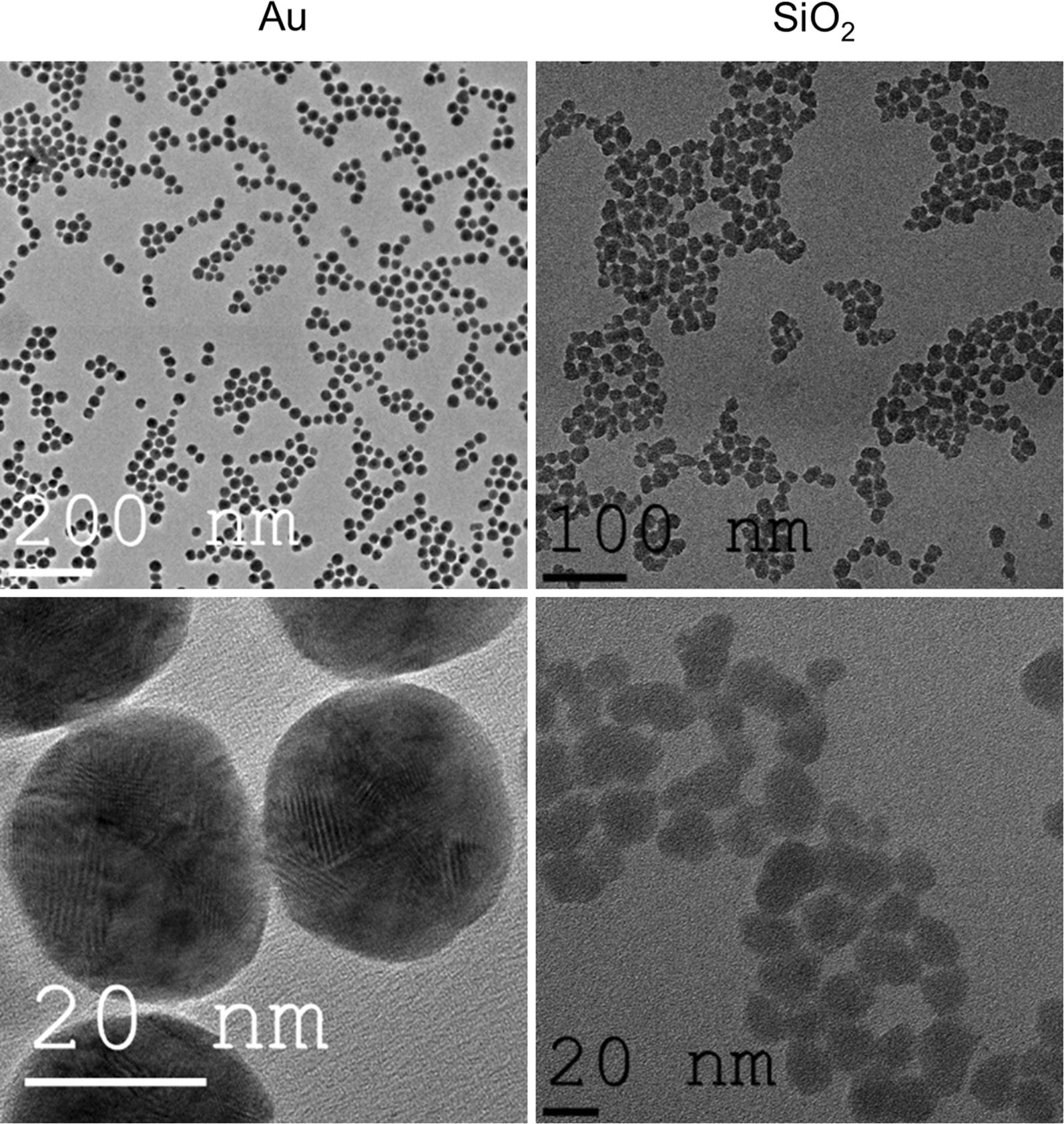
Representative transmission electron microscopy images of the Au (left) and SiO_2_ (right) NPs used in the present work

In the present study, commercially available gold and silicon dioxide NPs were used. The NPs had actual sizes of 25.3 and 20.4 nm, for gold and SiO_2_ respectively, as measured by transmission electron microscopy (TEM) (Fig. 1). In phosphate buffered saline, their hydrodynamic diameters were 39.8 and 35.7 nm, indicating high colloidal stability, which is likely bestowed by their negative surface charges of − 27.1 and − 35.4 mV, respectively. No endotoxins were found in the NP suspensions as determined by a LAL-assay. The small hydrodynamic diameter also suggests the high monodispersity of both samples and the lack of any substantial aggregation, which was further supported by the low polydispersity index (0.131 and 0.168, respectively). In the present work, the NPs were used to label two commonly used cell types, being human bronchial epithelial cells (BEAS-2B) and murine mesenchymal stem cells (MSCs).

### Nanoparticle-mediated cell death and oxidative stress

**Fig. 2:**
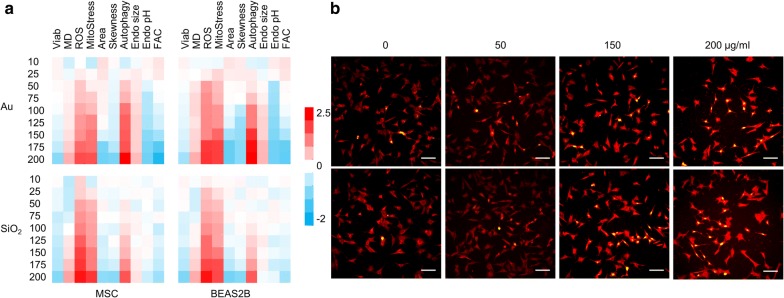
**a** Heat maps of the high content imaging data obtained for MSC (left), or Beas2B cells (right) exposed to various concentrations (10–200 µg/ml) of Au (top half) or SiO_2_ (bottom half) NPs for 24 h and analyzed for relative cellular health (Viab), membrane damage (MD), mitochondrial ROS (ROS), mitochondrial health (MitoStress), cell area (Area), cell skewness (Skewness), the level of autophagy, size of the endosomal network (Endo size), average endosomal pH (Endo pH) and total size of cellular focal adhesion complexes (FAC). Data are shown as relative values after *z*-normalization compared to untreated control cells (= 1) where the fold-change is indicated by the respective color-code. Data have been acquired for a minimum of 5000 cells/condition which were gathered from three independent experiments. **b** Representative high content images of MSCs either unlabeled (0 µg/ml) or labelled with Au (top row) or SiO_2_ (bottom row) NPs at the concentrations indicated for 24 h. Cells were stained with Live Dead dead cell stain (green) and MitoTracker Red CMXRos (red). Scale bars: 100 µm

As a first step, both cell types were exposed to a wide concentration range of either NP, after which a wide number of cellular parameters were evaluated using a validated high-content imaging based procedure [5, 6, 14]. Figure 2 reveals concentration-dependent loss of cell viability with is linked with an increase of cell membrane damage. Under our conditions, significant toxicity was only observed at concentrations of 200 µg/ml for both the Au and SiO_2_ NPs, in either of the cell types used. As no significant membrane damage was observed at subcytotoxic conditions, this suggests that the observed damage was a secondary effect of the dying process, rather than being a NP-induced mechanism, as has been observed for other NPs, such as hydrophobically-capped Au NPs [14, 32, 33]. At the highest subcytotoxic concentration, both NPs induced significant levels of oxidative stress, which is in line with literature data [34, 35]. Oxidative stress can cause a variety of secondary effects, but as mentioned above, is not always correlated with cell death, due to the cellular antioxidant capacities which can differ widely between different cell types [17]. Here, for both NP types, oxidative stress correlated nicely with the onset of mitochondrial stress, indicating cellular damage, which may eventually have led to the observed cell death at higher NP concentrations. Mitochondria are the main energy providers for the cell, but their metabolic processes such as oxidative phosphorylation also generate ROS, making them susceptible to additional forms of oxidative stress [36]. Stressed and damaged mitochondria can directly result in cell death, as mitochondrial leakage and release of cytochrome c is known to be a key component in the intrinsic apoptotic pathway [37]. Together, these data suggest that the oxidative stress and associated mitochondrial damage incurred by cellular NP internalization are likely the underlying causes of the observed cell death at higher NP concentrations.

### Nanoparticle-mediated effects on cellular autophagy and endosomal network

**Fig. 3:**
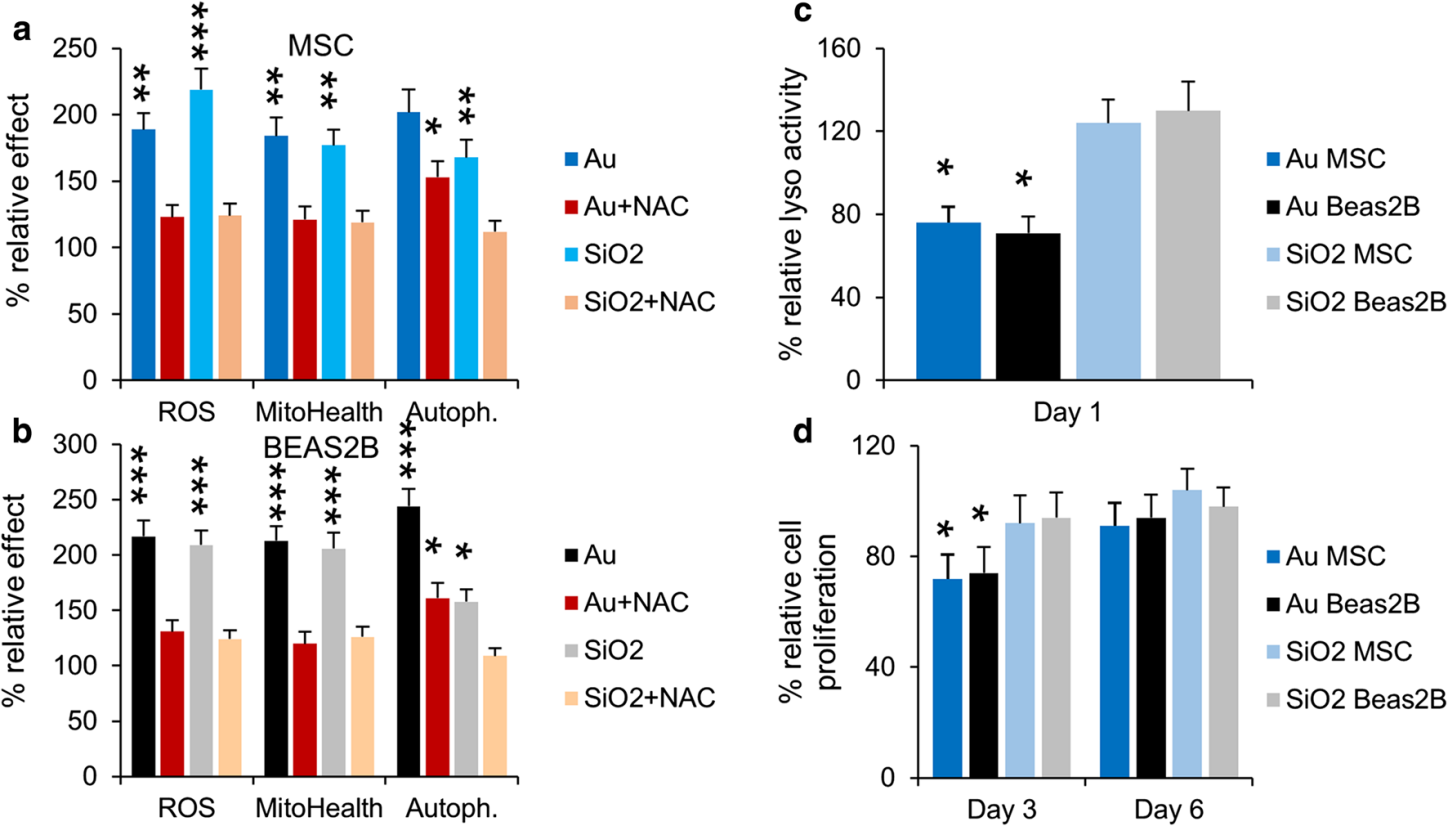
**a**, **b** Histograms representing the high content imaging data for **a** MSC and **b** Beas2B cells exposed to Au or SiO_2_ NPs at 150 µg/ml for 24 h (subcytotoxic conditions) in the absence or presence of 5 mM NAC, a free radical scavenger. The results for ROS, mitochondrial health and autophagy are presented relative to the level observed for untreated control cells. **c** Histograms representing the lysosomal activity of MSC and Beas2B exposed to Au or SiO_2_ NPs (150 µg/ml for 24 h) at 1 days after NP exposure. Data are expressed relative to the level for untreated control cells (100%). **d** Histograms representing the cellular proliferation of MSC and Beas2B exposed to Au or SiO_2_ NPs (150 µg/ml for 24 h) at 3 and 6 days post NP exposure. Data are expressed relative to the level for untreated control cells (100%). **a**–**d** Data are expressed as mean ± SD (*n* = 3). The degree of statistical significance is indicated when relevant (*p < 0.05; **p < 0.01; ***p < 0.001)

Apart from inducing apoptosis, damaged mitochondria may also induce autophagy in an attempt to self-preserve [38]. Figure 2 shows clear concentration-dependent increases in cellular autophagy levels. Interestingly, although the levels of oxidative stress and mitochondrial damage were similar for the Au and SiO_2_ NPs, the level of autophagy induction was more outspoken for Au NPs (Fig. 3a, b). When cells were treated with *N*-acetyl cystein (NAC), a ROS scavenger, the level of oxidative stress and mitochondrial damage were completely reduced to control levels for both Au and SiO_2_ NP-treated cells (Fig. 3a, b) like what has been observed for TiO_2_ based nanoparticles [39]. However, the level of autophagy was only completely reduced for SiO_2_ NP-treated cells, whereas Au NP-treated cells displayed a clear but not complete reduction of cellular autophagy levels. This finding suggests that the elevated autophagy levels for Au NP-treated cells were not solely caused by induction due to mitochondrial damage, but were also influenced by other factors. Other studies on gold NPs have revealed that they may induce an alkalizing effect on the endosomal system, which may reduce the functionality of the endosomes and hereby limit clearance of autophagosomes, resulting in an accumulation of autophagy-related vesicles [31]. To verify this, the size of the endosomal network and the average pH of the endosomes were monitored, showing no significant changes in either of these factors for cells exposed to SiO_2_ NPs. Au NP-treated cells however showed an enlarged endosomal network and an increase in endosomal pH (Fig. 2). These data suggest that the activity of the endosomes in which the Au NPs are localized may be affected. This was further confirmed by performing a lysosomal activity assay (Fig. 3c), which revealed a clear drop in lysosomal activity for Au NP-treated cells. From a mechanistic point of view, Au NPs could cause a loss of cellular degradative capacity by means of steric hindrance, where the persistent presence of non-biodegradable NPs within the endosomal network may diminish the overall degradative capacity of the cell. Alternatively, Au NPs have also been found to affect endosomal pH by interfering with the composition of the vacuolar H^+^(V)-ATPase, which regulates lysosome acidification. This ATPase is composed of a membrane-associated ion conductance Vo protein complex and a peripherally associated ATPase V1 protein complex, and Au NPs have been found to result in a dissociation of both components, hereby reducing its functionality [31]. The observed differences here between Au and SiO_2_ NPs suggests that the nature of the inorganic core plays a pivotal role in this alkalinization effect. This effect is likely to occur less for NPs that are prone to degradation (e.g. Ag, Fe_x_O_y_, ZnO, CuO) and even SiO_2_, which has also been found to degrade in an aqueous environment, albeit more rapidly under more alkaline conditions [40]. The impact of the coating of the NPs on these processes remains unclear, where the impact on differences in cellular uptake levels, intracellular distribution and persistence against biodegradation may all affect cellular degradation capacities.

Deactivation of the lysosomes will have an impact on the cellular degradative capacity, which can result in changes in cellular signaling, enhancing cellular autophagy levels in a manner to overcome the loss in degradative capacity. Interestingly, the activity of the lysosomes in SiO_2_ NP-treated cells was found to slightly increase (Fig. 3c). Lysosomal activation has been rarely reported for NP-treated cells, but has indeed already been observed for silica NP-exposed cells [41]. Lysosomal activation likely stems from the intrinsic nature of these organelles and the manner by which they deal with foreign pathogens. Changes in endosomal network have been described to stem from cellular conditioning to the presence of the NPs in an attempt to properly handle the influx of these foreign compounds [42].

### Nanoparticle-mediated effects on cellular cytoskeleton network

Autophagy has also been shown to affect other cellular processes, such as for instance, the tubulin network [43]. Analysis of cell size and skewness through staining of cytoskeletal networks revealed a clear loss of cytoskeletal organization, which resulted in shrinkage and shape-changes of cells exposed to the Au NPs at subcytotoxic conditions (Fig. 2). Interestingly however was that these alterations were not observed for SiO_2_ NP-treated cells (Fig. 2). Despite a difference in the degree of autophagy, SiO_2_ NPs still induced significant levels of autophagy, but this has no effect whatsoever on the cytoskeletal network. Previous studies have reported clear effects of various NPs including iron oxide, silver and gold on cell size and cytoskeletal network, predominantly affecting actin fibers [6, 44–47], while this effect was not observed for silica NPs [48]. Silica NPs have however been observed to affect tubulin fibers [49]. As silica NPs were also found to activate lysosomes [41], this may suggest an involvement of the lysosomal activity status on the precise nature of the cytoskeletal changes observed.

Both actin and tubulin network are involved in a wide array of cellular processes ranging from structural support to mediators in various intracellular signaling pathways [50]. The effect of any NPs on these networks may therefore have profound effects on various signaling pathways, which could turn into a loss of cell migration, cell differentiation or even result in cell death [45, 49]. One of the key mediators in cytoskeletal signaling are the focal adhesion complexes (FAC) which connect the actin cytoskeleton to transmembrane integrin receptors and transmit external signals through various protein complexes [51]. Here, we observed a significant loss of FAC for Au NP-treated cells, while no difference was observed for SiO_2_ NP-treated cells (Fig. 2). The functional implications of the loss of FACs were portrayed by the decrease in cell division rate for Au NP-treated cells, whereas no alterations in cell division times were observed for SiO_2_ NP-treated cells (Fig. 3d).

### Cellular alterations caused by lysosomal deactivation

**Fig. 4:**
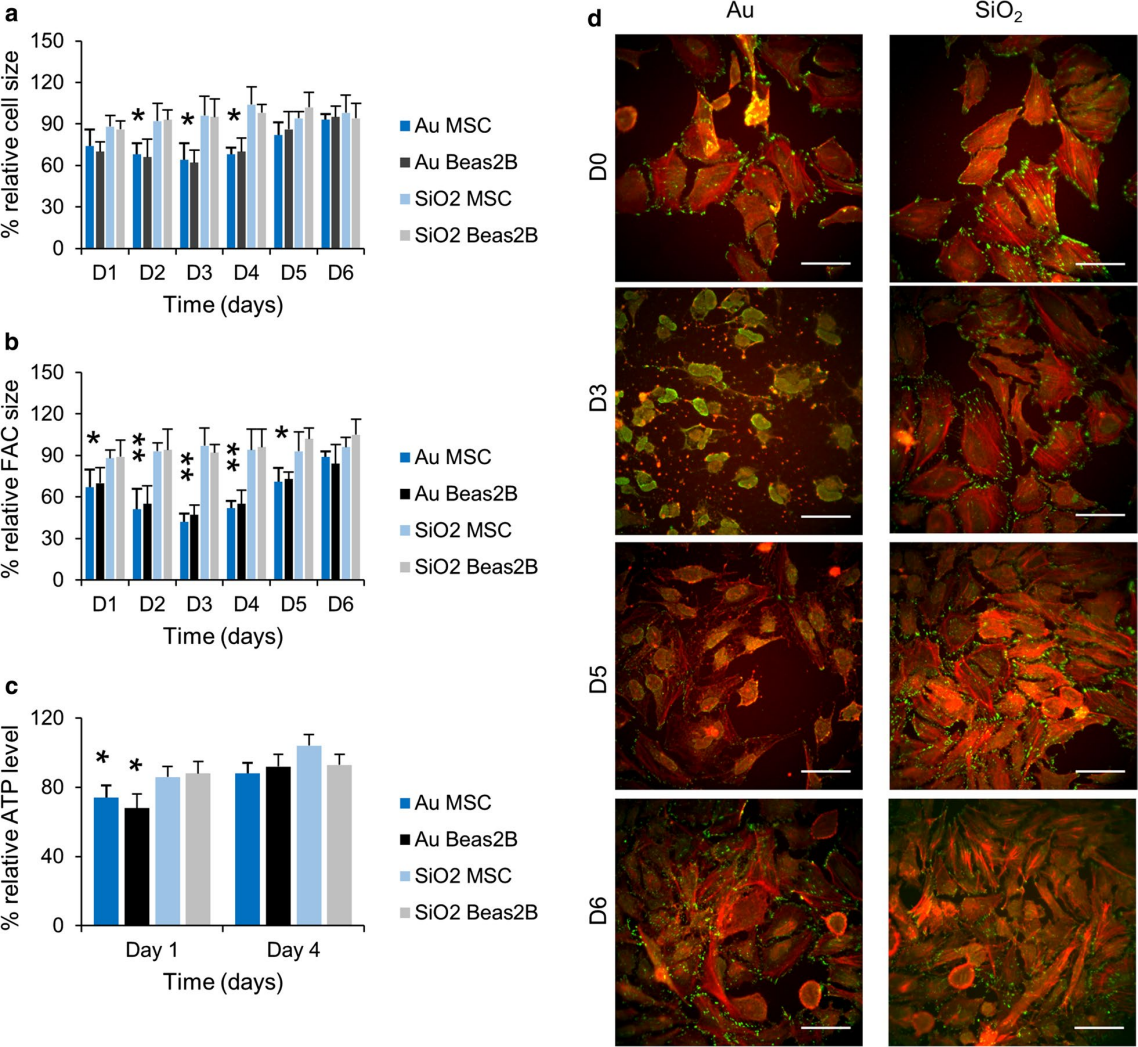
**a**, **b** Histograms representing high content imaging data for MSC and Beas2B cells exposed to Au or SiO_2_ NPs at 150 µg/ml for 24 h (subcytotoxic conditions). The data for **a** cell size and **b** FAC size are expressed as mean ± SD (*n* = 3) relative to the level for untreated control cells (100%). The results for ROS, mitochondrial health and autophagy are presented relative to the level observed for untreated control cells. **c** Histograms representing the cellular ATP levels of MSC and Beas2B exposed to Au or SiO_2_ NPs (150 µg/ml for 24 h) at 1 day and 4 days after NP exposure. Data are expressed as mean ± SD (n = 3) relative to the level for untreated control cells (100%). The degree of statistical significance is indicated when relevant (*p < 0.05; **p < 0.01). **d** Representative high content images of control MSC (D0) or MSC labelled with the Au or SiO_2_ NPs at 150 µg/ml for 24 h and then stained for actin (red) and vinculin (green), a marker for focal adhesion complexes 3, 5 and 6 days after labelling. Scale bars: 50 µm

Alterations to the cell size and shape have been frequently reported to accompany cellular NP uptake and have been associated to changes invoked by alterations required for NP endocytosis or through steric hindrance caused by the intracellular presence of large volumes of NPs sequestered in the endosomal compartment [44, 52]. To verify whether this is a transient phenomenon, the effect of the NPs on cell size was analyzed at several time points post cellular NP exposure. Figure 4a reveals that SiO_2_ NP did not have any effect at any time point, while the Au NPs resulted in significant but reversible effects. These effects were however delayed, where maximal effects occurred 2 days after NP exposure, while cells were nearly recovered after 5 days post NP exposure. The delayed effect suggests that the cytoskeletal changes were not inherent to the endocytic processes through which the cells took up the NPs, but rather hinted at transient cellular alterations. As SiO_2_ NPs did not cause any such effects, the elevated autophagy levels and loss of lysosomal activity seen for Au NP-treated cells may be involved. A near identical pattern was observed upon analysis of cellular FACs (Fig. 4b), where SiO_2_ NPs had only minimal effects, whereas Au NPs resulted in significant but transient reduction of total cellular FAC sizes. These data suggest that any functional effect observed will also be transient in nature. The latter may explain the high level of discrepancy observed for various NPs, mainly iron oxide NPs and their effect on stem cell differentiation. Various research groups have observed clear inhibition of chondrogenesis for iron oxide NP-labelled MSCs, while other groups saw no effect of the iron oxide NPs, even when identical commercial particles were used [53–56]. As the differentiation protocol takes rather long (up to 2 weeks), but can vary between different protocols used, the occurrence of any inhibition may be explained by the time window used and whether cells had sufficiently recovered from their transient functional impediments.

The mitochondrial stress incurred by the Au or SiO_2_ NPs may cause some metabolic disorders and reduce the overall cellular energy levels as mitochondria are the main sources of cellular ATP [36]. The induction of autophagosomes which can engulf the damaged organelles and then fuse to lysosomes to promote the degradation of their contents produces new metabolites that can be used as sources of energy [57]. Promoting the cellular degradation capacity, as observed for the PS NP-treated cells can therefore be seen as a cellular attempt to restore any loss in cellular energy levels. To test this hypothesis, cellular ATP levels were measured for SiO_2_ and Au NP-treated cells (Fig. 4c), which revealed a clear transient loss of cellular ATP for Au NP-treated cells, while no effect was noticed for SiO_2_ NP-treated cells. For Au NPs, the process of energy restoration appears to be flawed. The loss of degradative capacity impedes the fusion of the autophagosomes and lysosomes and thus cannot restore any ATP. The alkalizing effect of the Au NPs on the endosomes can further decrease cellular ATP levels as maintenance of lysosomal pH is an ATP-dependent process [57].

### Mechanisms underlying cellular changes through lysosomal deactivation

**Fig. 5:**
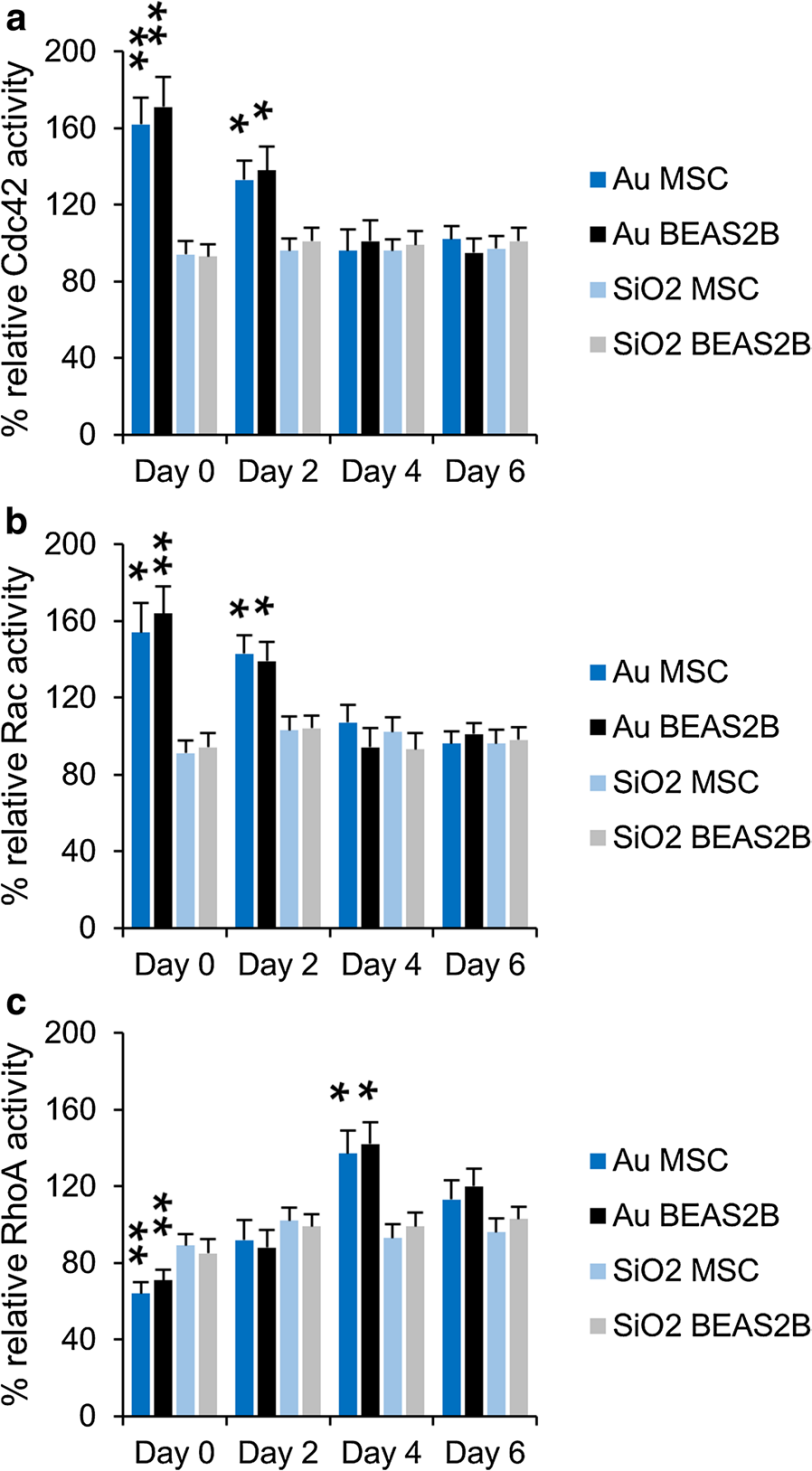
**a**–**c** Histograms representing **a** Cdc42, **b** Rac and **c** RhoA activity levels for MSC and Beas2B cells exposed to Au or SiO_2_ NPs at 150 µg/ml for 24 h (subcytotoxic conditions). The data are expressed as mean ± SD (*n* = 3) relative to the level for untreated control cells (100%) and are given for 0, 2, 4 and 6 days post NP exposure. The degree of statistical significance is indicated when relevant (*p < 0.05; **p < 0.01)

The loss of FACs, cytoskeletal deformations and decrease in cell division rates in Au NP-treated cells may be linked to the observed changes in cellular degradative capacity (autophagy induction) and cellular ATP levels. We tested the activity levels of both Cdc42, Rac and RhoA, which are important GTPases involved in cytoskeletal organization and cell cycle progression [58]. The data reveal that SiO_2_ NP-treated cells, as expected, did not show any significant changes in the activity status of either of the three GTPases (Fig. 5).

**Scheme 1:**
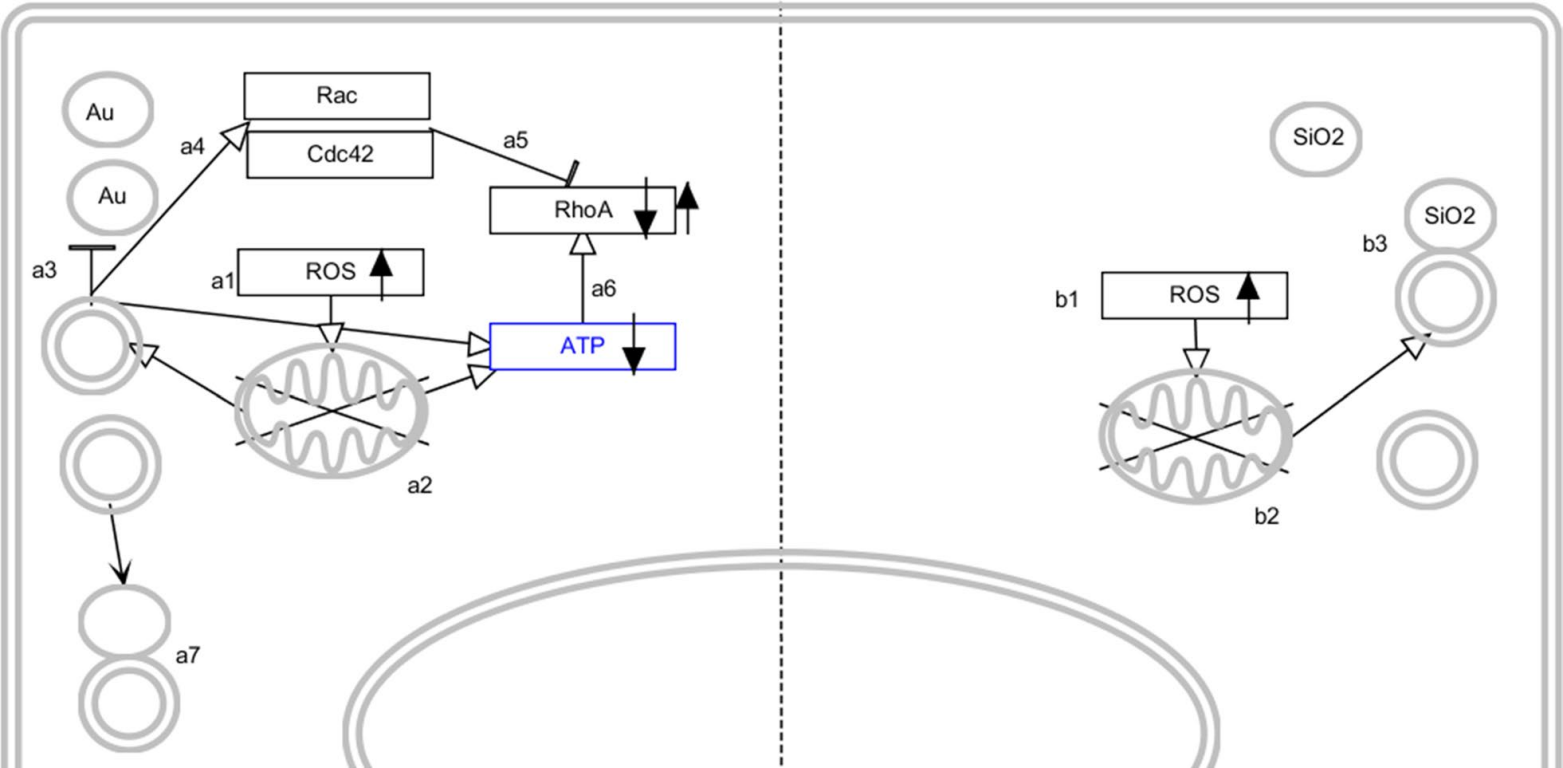
Schematic representation of the cellular effects of Au (left side) and SiO_2_ NP (right side) exposure. Au NPs enter the cells and induce ROS (a1) which causes mitochondrial damage (a2). This stimulates autophagy induction, but autophagosomes cannot be efficiently cleared due to the reduced lysosomal activity (a3), resulting in an accumulation of autophagosomes. This stimulates Rac and Cdc42 activity (a4), which affects cytoskeletal organization and actin-mediated signaling. Simultaneously, Cdc42 and Rac inhibit RhoA activity (a5). Mitochondrial damage and accumulation of autophagosomes lowers cellular ATP levels which in turn stimulates RhoA activity. Upon recovery of the cellular degradative capacity, autophagosome clearance occurs more efficiently (a7), which reduces cellular autophagy levels and Rac and Cdc42 activity. The low ATP levels then result in increased RhoA activity, which will return back to baseline levels as turnover of damaged mitochondria occurs more efficiently. RhoA activity will restore the cellular cytoskeleton and actin-mediated signaling. SiO_2_ NPs also enter the cells and induce ROS (b1), which affects mitochondrial health. This stimulates autophagy (b2) which promotes clearance of the damaged mitochondria. Upon fusion with the lysosomes, mitochondrial turnover remains normal, resulting in an efficient management of cellular ATP levels

Au NP-treated cells displayed clear activation of Cdc42 and Rac immediately after cell labeling while RhoA activity decreased. This is in line with the known functions of Cdc42 and Rac, which increase FAC turnover and hereby reduce the number of FAC. After 2 days, the activity levels of Cdc42 and Rac decrease to return to normal baseline levels after 4 days, while the activity of RhoA increases and is significantly elevated 4 days after cellular NP exposure, and then also returns to baseline levels at 6 days post NP exposure. These findings are perfectly in line with our observed kinetic alterations of the cellular cytoskeleton network. Based on known activators of the different GTPases, the following mechanism can be hypothesized (Scheme 1). The three GTPases are known to be carefully regulated to provide tight control over FAC turnover. Cdc42 and Rac activation increases FAC turnover while RhoA activation increases actin stress fiber formation and decreases FAC turnover [59]. Cdc42 and Rac have been described to be activated through autophagy induction [59], while their activation typically reduces RhoA activity [60]. As the cellular degradative capacity is reduced and autophagosomes cannot be efficiently cleared through lysosomal fusion, autophagy induction is likely to only be short-term, resulting in the decrease of Cdc42 and Rac activity levels with time. While Cdc42 and Rac activities go down, RhoA activity is able to rise and get back to normal levels. However, the low levels of cellular ATP triggers RhoA activity [61] and makes it significantly elevated above baseline levels. The restoration of cellular mitochondria and increase in ATP levels will finally stabilize RhoA activity back to baseline levels. The initial elevated Cdc42 and Rac activity will cause the cytoskeletal deformations and impede cell division, while the elevated RhoA activity at a later stage will restore FACs, actin stress fibers and cell cycle progression rates. Most effects observed here for the Au NP-treated cells are therefore transient ones that are related to the loss of lysosomal degradation capacity. Division of the cells and the associated dilution of the NPs amongst the two daughter cells helps the cells to recover and return to normal baseline physiological levels.

Overall, the Au NP-elicited effects are mainly caused by the precise chemical nature of the NP and the combination of several factors, being the induction of oxidative stress resulting in mitochondrial damage, the induction of autophagy and the loss in lysosomal activity inhibiting autophagosome clearance. These parameters must all occur under specific conditions which do not inflict any cell death and will therefore only happen with certain NPs under specific conditions that are capable of inducing the right level of oxidative stress, autophagy and impede lysosomal activity. The affected cellular signaling pathways can however have great implications for cell functionality, including loss of stem cell differentiation capabilities, cell division rates or cell migration properties which warrants their careful evaluation [44, 49].

### Functional effects caused by differences in cell signaling pathways

**Fig. 6:**
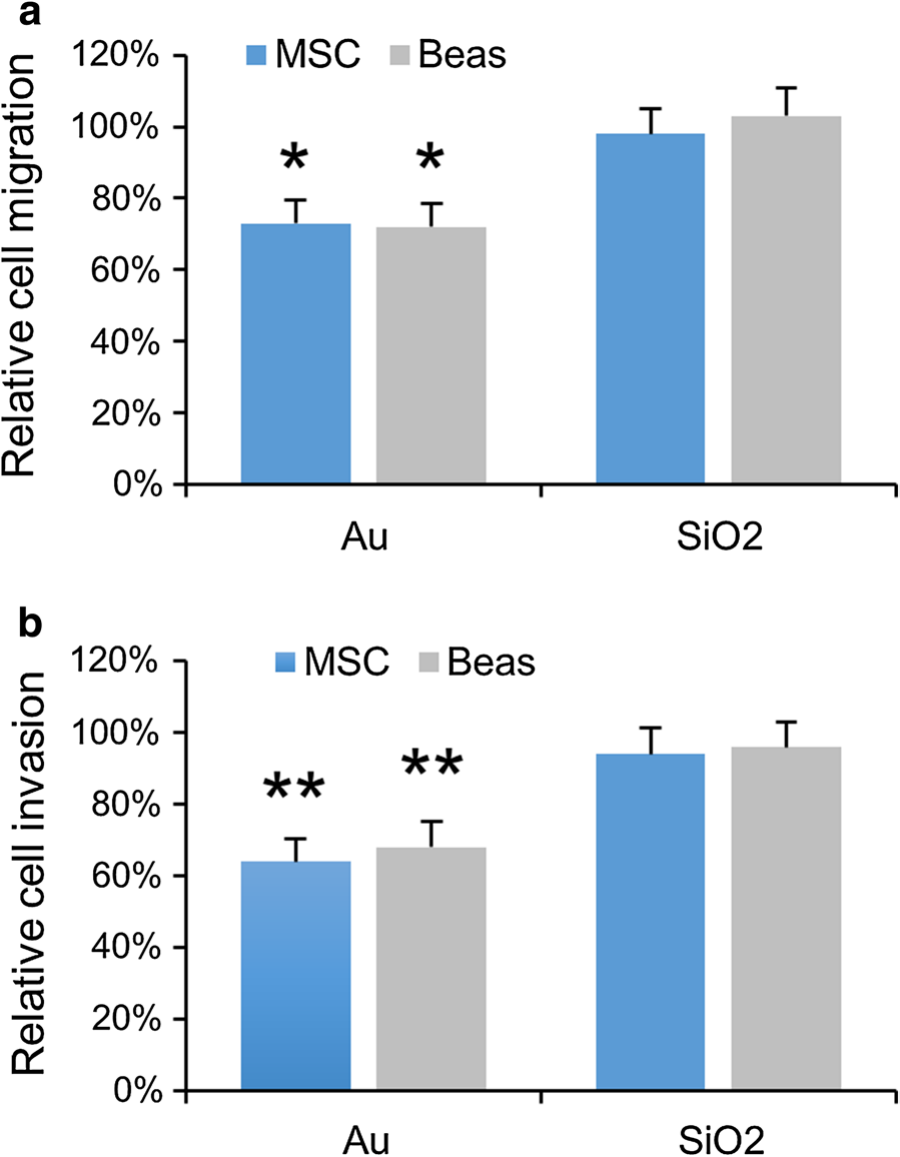
**a**, **b** Histograms representing **a** relative cell migration and **b** relative cell invasion levels for MSC and Beas2B cells exposed to Au or SiO_2_ NPs at 150 µg/ml for 24 h (subcytotoxic conditions). The data are expressed as mean ± SD (*n* = 3) relative to the level for untreated control cells (100%). The degree of statistical significance is indicated when relevant (*p < 0.05; **p < 0.01)

The affected activity of the GTPases can also result in losses of cellular functioning, as these GTPases are key signaling mediators in a broad number of cellular signaling pathways [58]. Here, the effect of the NPs on cell migration was studied, which is an important functional property for both cell types. For alveolar epithelial cells like Beas-2B, cell migration plays a pivotal role in airway repair and remodeling involved in respiratory diseases such as asthma [62], while MSC are, amongst others, the cellular source of fracture healing and are recruited to bone fracture sites [63]. Figure 6 shows that the SiO_2_ NPs did not have any significant effect on the migration or invasion efficacy of either the Beas-2B or MSC cells, while at subcytotoxic concentrations, the Au NPs significantly impeded both migration and invasion. These findings are completely in line with the differences in cytoskeletal alterations and affected cell signaling pathways. The reduced migration and invasion may be explained by the reduced ATP levels caused by the Au NPs, as low levels of cytoplasmic ATP reduce the function of vacuolar H^+^-ATPases, which has been shown to be associated with a reduction in cellular migration and invasion [64].

Together, these data indicate how relatively minor differences in the initial biological effects of both NP types can result in large differences in cellular functionality. This knowledge can be very useful for a wide range of biomedical applications. Firstly, if time permits, cells can be labelled with Au NPs and then used for functional studies when sufficient time has passed and cellular functionality has been restored. If the migratory capacity of the cells is important, Au NPs appear to be less suitable than SiO_2_ NPs. While both NPs are frequently being used as drug delivery vehicles, the nature of the drug may determine which type of NP would be better suited. For acid-labile agents that rapidly degrade under acidic conditions, the Au NPs may offer some protection through the alkalinization of the lysosomal lumen. Pro-apoptotic drugs may be better combined with SiO_2_ NPs, as the higher levels of autophagy for Au NPs may impede the pro-apoptotic signaling induced by the drugs. Autophagy-inducing anticancer drugs may then be more optimally suited for use with the Au NPs, where the pro-autophagy effects of both the carrier and the drug can be combined.

## Conclusions

The present work highlights the importance of a full comprehension of bio-nano interactions to explain any observed functional behavior in biological components. Here, gold and SiO_2_ NPs had rather similar toxicity profiles, where they differed in one seemingly minor parameter, being the lysosomal activity. However, coupled with the other cellular alterations, including mitochondrial ROS and autophagy induction, this parameter became very important. For SiO_2_, lysosomal activity slightly increased, enabling an efficient clearance of autophagosomes and recycling of autophagy-processed cellular materials, such as mitochondria. This led to an efficient clearance of damaged mitochondria and replacement by new ones. For Au NPs, lysosomal activity was decreased, which impeded clearance of the autophagosomes. This led to the activation of Rac and Cdc42, which affect cytoskeletal organization and actin-mediated signaling, slowing down cell division. This also resulted in a less efficient turnover of the damaged mitochondria, resulting in a loss of ATP, which activated RhoA signaling. Initially, Rac and Cdc42 activity inhibited RhoA activity, but upon recovery of the cellular degradative capacity, and decrease of Rac and Cdc42 activity, RhoA activity became more dominant. This resulted in a recovery of the baseline cytoskeletal architecture and actin-mediated signaling levels. Simultaneously, the increased cellular degradative capacity increases the turnover of damaged mitochondria and restores cellular ATP levels, which finally reduces RhoA activity back to baseline levels. These data reveal that a minor difference in biological impact of NPs can, in combination with other toxicological effects, result in a wide range of altered signaling pathways which can have a broad range of functional implications. In the present study, the clear impact on cytoskeletal alterations was easily noticed, but more subtle differences can often be overlooked. Here, the effects were only transient, indicating the need for more kinetic studies to further elucidate the impact of NPs on biological components.

## Experimental section

### Nanoparticles and characterization

Gold and silica NPs were obtained commercially via NanoComposix, Ltd (product numbers AULB20-5M and SISN20-25M, respectively). The gold NPs were provided with lipoic acid, while silica NPs had free silanol groups at their surface. The purchased NPs were both 20 nm diameter according to the company.

In-house characterization was performed, including analysis of size [transmission electron microscopy (TEM)], hydrodynamic size and surface charge (dynamic light scattering) and sterility tests (LAL-endotoxin assay). For the TEM specimen preparation, a drop of nanoparticle solution was dropped on the Cu-grid with C-film for the TEM investigation. All the grids with the sample were dried at RT followed by scanning the large regions of the grid. The low and high resolution TEM of the sample were examined using transmission electron microscopy (TEM) on a FEI Titan 80/300 microscope equipped with a Cs corrector for the objective lens, a Fischione high angle annular dark field detector (HAADF), GATAN post-column imaging filter and a cold field emission gun operated at 300 kV as an acceleration voltage. Electrophoretic mobilities and hydrodynamic radii were measured with a Zetasizer ZS90 instrument (Malvern, UK) at 25 °C. To optimise the response for every given sample, the samples were diluted with PBS (1/100) immediately prior to the measurement. The LAL-assay was performed according to the manufacturer’s protocol (Pierce).

### Cell culture

Human bronchial epithelial cells (BEAS-2B) were grown in high glucose containing Dulbecco’s modified Eagle’s medium (DMEM), supplemented with 10% fetal calf serum, 1 mM sodium pyruvate, 2 mM l-Glutamine and 1% penicillin/streptomycin (Gibco, Invitrogen, Belgium). The BEAS-2B cells were passaged upon reaching 80% confluence and reseeded at a ratio of 1:5.

Mouse mesenchymal stem cells (MSCs) were maintained in high glucose containing Dulbecco’s modified Eagle’s medium (DMEM), supplemented with 10% fetal calf serum, 10% horse serum, 1 mM sodium pyruvate and 2 mM l-Glutamine (Gibco, Invitrogen, Belgium). Cells were passaged when reaching nearly 80% confluence and reseeded at a density of 100,000 cells/flask in 75 cm^2^ tissue culture flasks (Nunc, Belgium).

### High-content analysis of cell-nanoparticle interaction studies

For high-content imaging studies, both cell types were seeded at 7500 cells/well in a 24 well plate (Nunc, Belgium). Cells were allowed to attach overnight in a humidified atmosphere at 37 °C and 5% CO_2_, after which the cells were incubated with either the Au or PS NPs for 24 h in full growth medium. For cellular exposure studies, cells were incubated with the NPs at 10, 25, 50, 75, 100, 125, 150, 175 and 200 µg/ml. Each experiment was performed in three independent repeats and data were analyzed using full data sets of the different repeats. The different assays were performed as described elsewhere [5, 14].

#### Heat maps

The generation of heat maps was performed using conditional formatting of Excel sheets after *z*-normalization of all the data. Data are expressed as the fold increase of a certain parameter (cell death, membrane damage, mitochondrial ROS, mitochondrial stress, cell area, cell skewness, autophagy levels, endosomal size, endosomal pH, focal adhesion complexes) compared to the control levels where the level of increase is indicated by colour-codes.

### Evaluation of RhoA, Cdc42 and Rac1 activity

MSC and Beas-2B cells were seeded in 25 cm^2^ collagen-coated tissue culture flasks at 1 * 10^5^ cells/flask and allowed to settle overnight. Next, cells were incubated with fresh media (10 ml) containing the Au or SiO_2_ NPs at 150 µg/ml for 24 h. Media were removed, cells washed twice with ice-cold PBS, and cells were then either kept in culture for an additional 2, 4 or 6 days or processed immediately. At these time points, cell lysates and GTPase activities were prepared and measured according to the manufacturer’s instructions (RhoA, Rac1, Cdc42 G-LISA activation kits, Cytoskeleton Inc, Denver, USA). Absorbance was recorded at 450 nm with an ELISA plate reader (Optima FluoStar, BMG LabTech GmbH, Ortenberg, Germany). For all three cell types, the results obtained for the NP-treated samples were normalized against the value obtained for untreated control cells at identical protein levels. Values are expressed as relative to those obtained for untreated controls cells (= 1) for a total number of three independent repeats.

### Measurement of cellular ATP levels

MSC and Beas-2B cells were seeded in 25 cm^2^ collagen-coated tissue culture flasks at 1 * 10^5^ cells/flask and allowed to settle overnight. Next, cells were incubated with fresh media (10 ml) containing the Au or SiO_2_ NPs at 150 µg/ml for 24 h. Media were removed, cells washed twice with ice-cold PBS, and cells were then either kept in culture for an additional 4 days or processed immediately. To lyse the cells, 1 ml of ice-cold lysis buffer was added per flask (100 mM Tris + 4 mM EDTA, pH 7.5) after which the lysates were processed according to the manufacturer’s instructions (ATP Determination kit, Thermo Fisher Scientific, Waltham, MA, USA). Luminescence was measured using the IVIS Spectrum well plate option 10 min after addition of d-luciferin. For all three cell types, the results obtained for the NP-treated samples were normalized against the value obtained for untreated control cells at identical protein levels. Values are expressed as relative to those obtained for untreated controls cells (= 1) for a total number of three independent repeats.

### Lysosomal activity measurements

MSC and Beas-2B cells were seeded in 25 cm^2^ collagen-coated tissue culture flasks at 1 * 10^5^ cells/flask and allowed to settle overnight. Next, cells were incubated with fresh media (10 ml) containing the Au or SiO_2_ NPs at 150 µg/ml for 24 h. Media were removed, cells washed twice with ice-cold PBS, after which cell lysates were prepared and acid phosphatase activity measured according to the manufacturer’s instructions (Acid Phosphatase assay kit, Sigma-Aldrich, St. Louis—MO, USA). For all three cell types, the results obtained for the NP-treated samples were normalized against the value obtained for untreated control cells at identical protein levels. Values are expressed as relative to those obtained for untreated controls cells (= 1) for a total number of three independent repeats.

### Cell migration and invasion

Both cell types were seeded in 25 cm^2^ collagen-coated tissue culture flasks at 1 * 10^5^ cells/flask and allowed to settle overnight. Next, cells were incubated with fresh media (10 ml) containing the Au or SiO_2_ NPs at 150 µg/ml for 24 h. Media were removed, cells washed twice with ice-cold PBS, after which they were reseeded in new 24 well plates at a density of 1 * 10^4^ cells/well, either the Radius™ 24 well cell migration assay plate (Cell Biolabs Inc, San Diego, CA, USA) or a 8 µm-pore Boyden chamber (Cell Biolabs Inc, San Diego, CA, USA). After 24 h, the gel plug was removed from the Radius migration assay plate, allowing the cells to migrate. To promote migration of the Beas-2B cells, they were exposed to 20 ng/ml IL-6. For cell invasion studies, cell media were removed and fresh serum-free media was given to the cells in the upper compartment, while to lower compartment contained full serum-containing medium. For Beas-2B cells, the lower compartment was also supplemented with 20 ng/ml IL-6. Cell migration was measured fluorometrically after 12 h, by fixing the cells, staining with PI and using the imaging shield which only allows light from the original gel plug-covered area to be measured. For cell invasion, the assay procedure was performed in accordance with the manufacturer’s instructions.

### Statistical analysis

All data are expressed as the mean ± standard deviation (SD). For all experiments, any statistical significance between a single condition and untreated control cells were analyzed using one-way ANOVA followed by a Dunnett post hoc test using Graphpad 6.
